# BMSCs reduce rat granulosa cell apoptosis induced by cisplatin and perimenopause

**DOI:** 10.1186/1471-2121-14-18

**Published:** 2013-03-19

**Authors:** Jun-qi Guo, Xia Gao, Zhi-jie Lin, Wei-zhen Wu, Liang-hu Huang, Hui-yue Dong, Jin Chen, Jun Lu, Yun-fen Fu, Jin Wang, Yu-jie Ma, Xiao-wen Chen, Zhi-xian Wu, Fu-qiang He, Shun-liang Yang, Lian-ming Liao, Feng Zheng, Jian-ming Tan

**Affiliations:** 1 Organ Transplant Institute, Fuzhou General Hospital, DongFang Hospital of Xiamen University and Fujian Key Laboratory of Transplant Biology, Fujian, 350025, PR. China

**Keywords:** Bone marrow mesenchymal stem cells, Granulosa cells, Apoptosis, Ovary, Rat, Perimenopause

## Abstract

**Background:**

The objective of this study was to evaluate the effect of bone marrow mesenchymal stem cells (BMSCs) on the apoptosis of granulosa cells (GCs) in rats.

BMSCs and GCs were isolated from rats. GCs were separated into one of the following three groups: an untreated control group (control), a cisplatin (5 mg/L) treatment group (cisplatin), and group co-cultured with BMSCs and treated with cisplatin (BMSC). GC apoptosis was analyzed by annexin V staining and real-time PCR analysis for apoptosis-related genes. The effect of BMSCs was also determined in 9 to 10 month-old perimenopausal rats that were separated into the following groups: saline control, BMSC transplantation (1–2 × 10^6^ cells), and estrogen treatment (0.158 mg/kg/d) groups. A young group consisting of 3 to 4 month-old rats that were treated with saline was also evaluated as a control. After 1 and 3 months, GC apoptosis was evaluated by TUNEL analysis.

**Results:**

Cisplatin increased GC apoptosis from 0.59% to 13.04% in the control and cisplatin treatment groups, respectively, which was significantly reduced upon co-culture with BMSCs to 4.84%. Cisplatin treatment increased p21 and bax and decreased c-myc mRNA expression, which was reversed upon co-culture with BMSCs. As compared to young rats, increased apoptosis was observed in the perimenopausal rats (*P* < 0.001). After 3 months, the apoptosis rate in the BMSC group was significantly lower than that of the control group (*P* = 0.007).

**Conclusions:**

BMSC therapy may protect against GC apoptosis induced by cisplatin and perimenopause. Further studies are necessary to evaluate therapeutic efficacy of BMSCs.

## Background

Perimenopause or the permanent end of menstruation and fertility is characterized by reduced estrogen levels and disorder of the hypothalamus-pituitary-ovary/adrenal axis. Therefore, estrogen replacement therapy is commonly prescribed to increase the quality of life for individuals in perimenopause. However, significant risks have been associated with estrogen replacement therapy, including increased risk of breast cancer, endometrial cancer, and cardiovascular events [1-3]. Thus, better treatments without the associated risks are required.

Granulosa cells (GCs) are responsible for estrogen synthesis within the ovary and subsequent follicular atresia, and their numbers decrease in perimenopause [4]. Therapies that prevent their apoptosis may represent a novel treatment modality. Previous studies have shown that mesenchymal stem cells (MSCs), including those derived from bone marrow (BMSCs), are capable of repairing a variety of injured tissues [5,6] due to their potential to differentiate into osteoblasts, chondrocytes, adipocytes, cardiocytes, and neural cells [7]. In rats with chemotherapy-induced ovarian damage, BMSC transplantation improved ovarian structure and function [8]. However, little is known about the therapeutic effects of BMSCs on GC apoptosis associated with perimenopause.

The objective of this study was to evaluate the effect of BMSCs on the apoptosis of ovarian GCs in perimenopausal rats. BMSC and GC cultures were isolated from rats and characterized using microscopy, immunocytochemistry, and flow cytometry. In vitro GC apoptosis was induced by cisplatin in GCs co-cultured with and without BMSCs. In addition, GC apoptosis was analyzed in the perimenopausal rats treated with BMSCs and estrogen using TUNEL assays and compared to untreated controls and young rats. BMSC therapy may represent a novel treatment for perimenopause that may have less deleterious effects than estrogen replacement therapy.

## Results

### Characterization of cultured BMSCs and GCs

**Figure 1:**
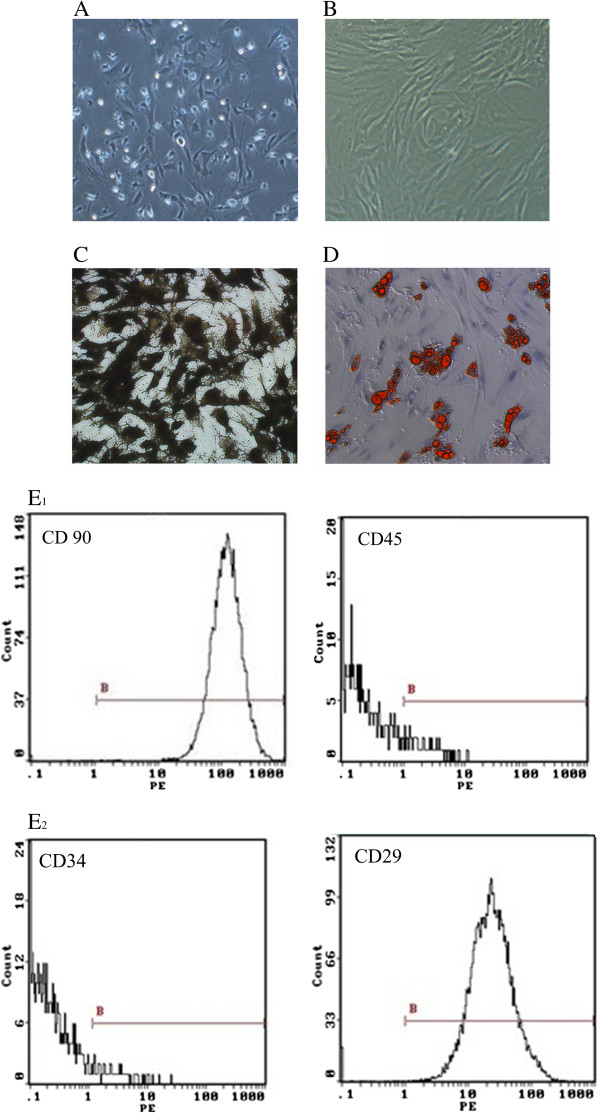
**BMSC morphology and surface marker expression.** BMSC morphology and differentiation was assessed by microscopy of BMSCs (**A**) in the first passage, (**B**) third passage, (**C**) after Von Kossa staining for visualization of osteoblasts after induction, (**D**) and after Oil red staining for adipocytes after induction (200×). (**E**) CD90, CD45, CD34, and Cd29 surface market expression was determined using flow cytometry.

Isolated BMSCs in culture were characterized by microscopy and flow cytometry analyses. Freshly isolated and cultured BMSCs were adherent, with most displaying polygonal morphology (Figure 1A). By the third passage, BMSC morphology resembled fibroblasts with few contaminant cells (Figure 1B). Most of the cultured BMSCs expressed CD29 (99.1% positive) and CD90 (98.2% positive) but were negative for markers of the hematopoietic lineage, including CD34 (1.01% positive) and CD45 (1.32% positive) (Figure 1E-H), which is consistent with previous studies [9,10].

To determine the extent to which induction resulted in BMSC differentiation toward osteoblasts versus adipocytes lineages, cells were examined after Von Kossa staining. As shown in Figure 1C (black cells) and 1D (red cells), isolated BMSCs were capable of differentiating into both osteoblasts and adipocytes. Furthermore, the basal secretion of large amounts of angiogenic and antiapoptotic factors from BMSCs cells after three days in culture was observed and included 1674.22 ± 182.80 pg/mL vascular endothelial growth factor (VEGF), 22255.75 ± 3473.66 pg/mL hepatocyte growth factor (HGF), and 0.9237 ± 0.13 ng/mL insulin-like growth factor-1 (IGF-1).

**Figure 2:**
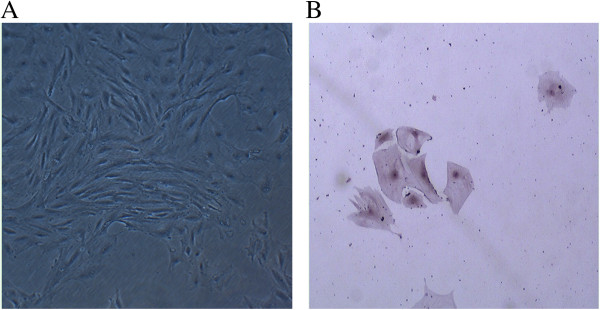
**GC morphology and FSHR expression.** (**A**) Primary GC morphology was assessed by microscopy. (**B**) Intracellular expression of FSHR by GCs was observed by immunocytochemistry (400×).

Isolated GCs were also characterized by microscopy and immunocytochemistry. Cultured GCs were adherent and displayed polygonal morphology (Figure 2A). Cultured GCs also expressed intracellular follicle stimulating hormone receptor (FSHR), confirming their identity as GCs (Figure 2B).

### Effects of BMSCs on GC apoptosis in vitro

**Figure 3:**
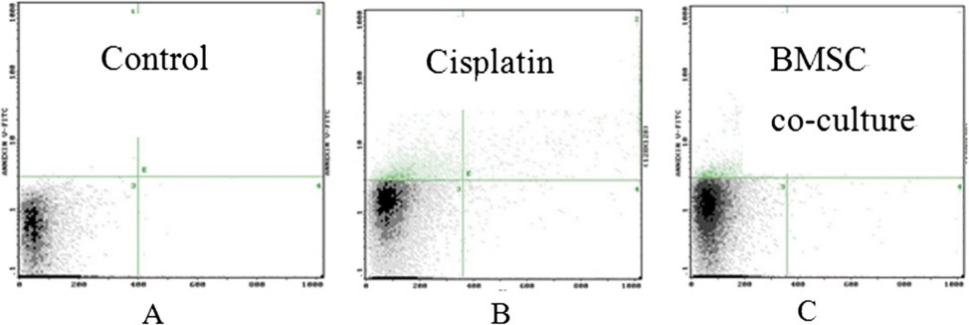
**Effects of BMSCs on GC apoptosis in vitro.** The apoptosis rate of GCs in the control (**A**), cisplatin (**B**) and BMSC co-culture (**C**) groups.

To determine whether BMSCs could protect GCs against cisplatin-induced apoptosis, GCs cultured in the absence or presence of BMSCs were treated with cisplatin. As shown in Figure 3, cisplatin increased apoptosis from 0.59% to 13.04% in the control and cisplatin groups, respectively (Figure 3A and 3B). However, co-culture with BMSCs significantly reduced GC apoptosis to 4.84% (Figure 3C).

**Figure 4:**
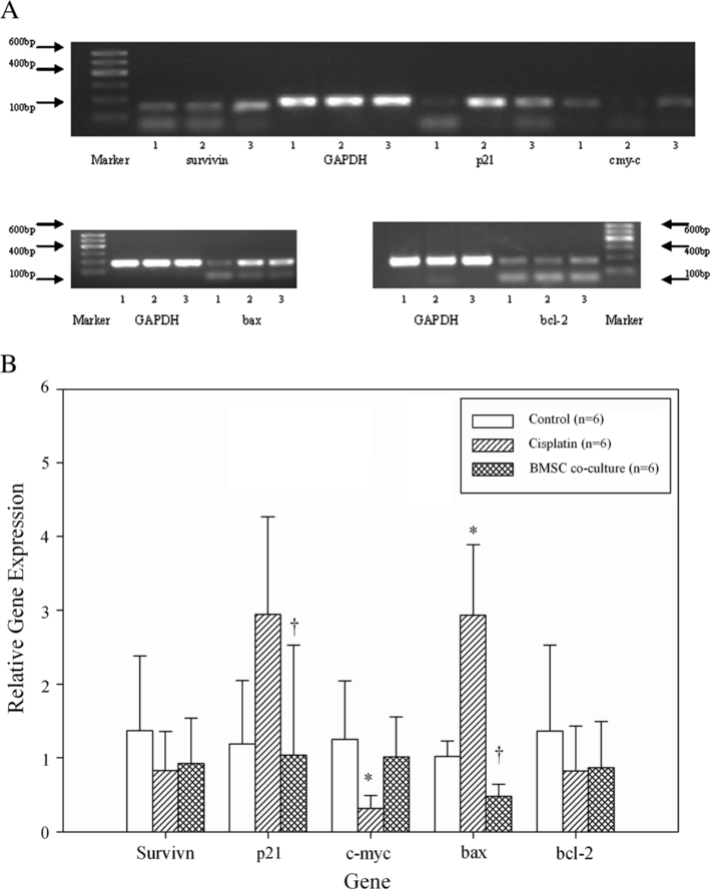
**Effects of BMSCs on apoptosis-related genes in GCs.** Relative gene expression levels were determined using real-time PCR among the control (lanes 1), cisplatin (lanes 2), and BMSC co-culture (lanes 3) groups. (**A**) Representative images for each gene analyzed were shown. (**B**) Relative gene expressions levels by using real-time PCR. ^*^ indicates a significant difference from the control group; *P* < 0.05, ^†^ indicates a significant difference from the cisplatin group; *P* < 0.05. N = 6 for each group.

Real-time PCR analysis was next undertaken to determine the effect of BMSC co-culture on the mRNA expression of apoptosis-related genes (Figure 4, N = 6 for each group). Although no significant changes in survivin and bcl-2 mRNA expression were observed among the three treatment groups, differences in p21 (*P* = 0.033), c-myc (*P* = 0.029), and bax (*P* < 0.001) mRNA expression were detected. Co-culture of GCs with BMSCs significantly decreased p21 mRNA expression (2.944 ± 1.322 vs. 1.034 ± 1.492, *P* = 0.046). Cisplatin treatment decreased c-myc mRNA expression as compared to the control group (1.253 ± 0.789 vs. 0.314 ± 0.177, *P* = 0.028), and this effect was ameliorated with BMSC co-culture. Furthermore, a significant increase in bax mRNA expression was observed with cisplatin treatment as compared to the control group (2.934 ± 0.955 vs. 1.019 ± 0.211, respectively; *P* < 0.001); however, it significantly decreased upon co-culture with BMSCs (0.476 ± 0.167, *P* < 0.001; Figure 4).

### Effects of BMSCs in an in vivo model of perimenopause

**Figure 5:**
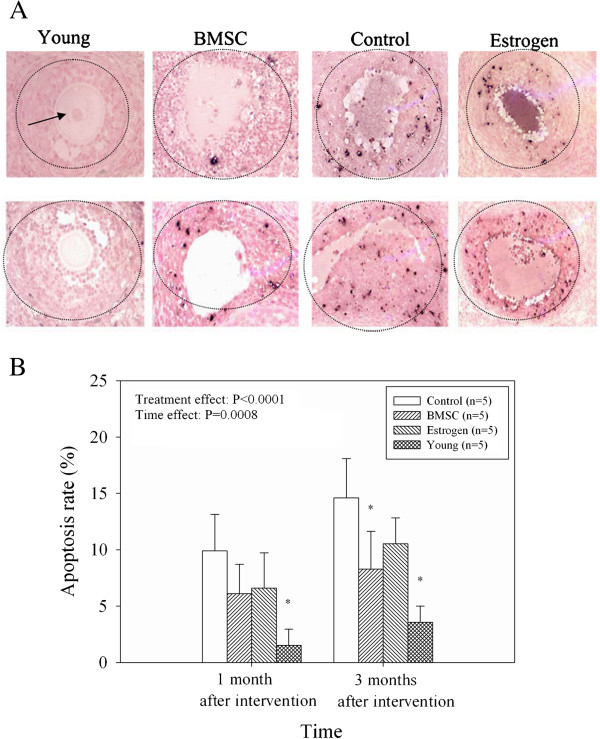
**Effects of BMSCs in an in vivo model of perimenopause.** GC apoptosis was assessed by TUNEL staining. (**A**) Representative images of ovarian tissues obtained from the young, BMSC, control, and estrogen groups one (top panels) and three (bottom panels) months after the intervention are shown (200×). (**B**) GC apoptosis rates among the control, BMSC, estrogen, and young groups after 1 and 3 months intervention. Values are presented as mean ± SD. ^*^ indicates a significant difference from control; *P* < 0.05 (Two-way ANOVA: treatment effect, *P* < 0.0001; time effect, *P* = 0.0008). N = 5 for each group.

To determine if BMSC transplantation influences the GC apoptosis associated with perimenopause, an in vivo model was established in middle-aged rats (Figure 5, N = 5 for each group). Both treatment (*P* < 0.0001) and time (*P* = 0.0008) had significant effect on the apoptosis rates. However, the interaction of time and treatment on the apoptosis rates was not significant (*P* = 0.638). As compared young rats, increased apoptosis was observed in the middle-aged rats after one month (1.53 ± 1.44% vs. 9.90 ± 3.23%, *P* < 0.001; Figure 5). After intervention with BMSC therapy or estrogen for 1 month, the apoptosis rate was lower than the control group although a significant difference was not observed. After 3 months, the apoptosis rate in the BMSC group (8.29 ± 3.35%, *P* = 0.007) was significantly lower than that of the control group (14.60 ± 3.51%). No difference in estrogen treatment was observed.

## Discussion

In the present study, co-culture with BMSCs significantly reduced cisplatin-mediated GC apoptosis and cisplatin-induced changes in p21, bax, and c-myc mRNA expression. In an in vivo model of perimenopause, the GC apoptosis rate was significantly lower in rats receiving BMSC therapy as compared to the control group after three months.

BMSCs account for 1–10 per 100,000 nucleated cells within bone marrow [11]. Although their isolation is relatively simple and they amplify in abundance in vitro, an ideal marker of BMSCs has yet to be identified. In this study, BMSCs expressed CD29 and CD90, but not CD34 and CD45, which is in agreement with Stagg et al. [9]. Characterization of their differentiation potential confirmed their ability to differentiate into osteoblasts and adipocytes in vitro. In addition, BMSCs can differentiate into chondrocytes, tenocytes, and neural cells [7].

Cisplatin induces DNA crosslinking, which triggers apoptosis. In the present study co-culture with BMSCs reduced GC apoptosis induced by cisplatin, which is in agreement with a previous study [8]. Also, co-culture of GCs with BMSCs downregulated the mRNA expression of Bax and p21 and upregulated c-myc mRNA expression in GCs. Bcl-2 and Bax are both apoptosis regulating proteins that effect the mitochondria [12,13]; Bcl-2 competes with Bax for interaction with adenine nucleotide translocator (ANT) on the mitochondrial membrane, preventing apoptosis [14]. In addition, p21 overexpression may promote apoptosis [15-17]; it regulates the cell cycle through p53-dependent and independent pathways [18]. Finally, c-myc promotes cell cycle progression from the G0/G1 phase to the S phase [14]; its downregulation has been associated with Bax oligomerization and subsequent activation of caspase-9 and caspase-3, resulting in apoptosis [19]. Because BMSCs and GCs were separated by a semipermeable membrane, protection by BMSCs was elicited through secreted paracrine factors. However, further studies are necessary to identify the precise paracrine mediator(s) responsible for this protective effect.

Perimenopause is defined as occurring 12 months after the last menstrual period. At present, there are four animal models of perimenopause, including an ovarectomized animal model, a model that physically or pharmacologically disrupts of ovarian function, a 4-vinylcyclohexene diepoxide (VCD) follicle depletion model, and a natural aging animal model. The natural aging animal model was selected for this study as it most closely models the pathological changes associated with perimenopause.

BMSC transplantation reduced GC apoptosis, which is in accordance with two previous studies describing the restorative effects of MSCs after their transplantation into ovaries [8,20]. However, the mechanism by which the BMSCs influenced GC apoptosis remains unclear as they were introduced via injection into vena caudalis. For example, the transplanted BMSCs may have reached the site of injury within the ovary and replaced damaged tissue through integration into the ovarian tissue and differentiation into the required cell type. Alternatively, the BMSCs may have induced tissue repair and prevented apoptosis by secreting paracrine mediators, including glial cell-derived neurotrophic factor (GDNF), brain-derived neurotrophic factor (BDNF), tumor necrosis factor (TNF), basic fibroblast growth factor (bFGF) [11,21-23], promoting functional recovery [24]. The latter mechanism is supported by the in vitro study that showed reduced GC apoptosis with BMSC co-culture, in which the cells were not in direct contact. Furthermore, analysis of cell culture supernatant revealed that BMSCs secreted IGF-1, HGF, and VEGF-A. VEGF promotes the formation of capillary networks, ensuring nutrition for GCs and oocytes [25]. In the ovary, IGF-1 regulates DNA replication of theca cells and GCs; it also regulates aromatase activity, secretion of inhibin, and generation of corpus luteum stimulating hormone receptor [26]. IGF-1 also prevents cell cycle progression from G0/G1 to S via PI3K/Akt signaling and inhibits Fas receptor-mediated apoptosis [27]. Finally, GCs express the HGF receptor, c-met [28]. Uzumcu et al. [29] reported that the level of HGF was inversely proportional to GC apoptosis, and HGF can reduce GC apoptosis in vivo and vitro. Further studies will be undertaken to elucidate the mechanism by which BMSCs influence GC apoptosis.

The present study has some limitations. For example, basic characterization of the perimenopausal rats was using vaginal smears to detect estrous cycle, estrous cycle disorders, and prolongation. However, the effects of perimenopause, estrogen therapy, or BMSC therapy on ovarian hormone levels in the serum were not determined. In addition, the in vivo study lacked a BMSC-only treatment group. Furthermore, the protein levels of c-myc, p21, survivin, Bcl-2, and Bax were not assessed to corroborate the data obtained using RT-PCR. Finally, the mechanism by which BMSCs influence GC apoptosis (i.e., integration and/or paracrine mediators) remains unclear and will be the subject of further studies.

## Conclusion

BMSCs decrease the GC apoptosis in vitro and vivo. Further studies will evaluate the therapeutic efficacy of BMSC therapy for perimenopausal symptoms as well as the basis for these effects.

## Methods

### Experimental animals

Immature male (60–80 g), young female (3–4 months), and perimenopausal female (9–10 months) Sprague–Dawley rats were obtained from the Animal Center at Shanghai. All rats were kept under controlled temperature (30 ± 2°C) and light (14 h light, 10 h dark) conditions with rat chow and water ad libitum. All experiments were approved by the institutional animal committee of the Fuzhou General Hospital.

### Isolation and identification of BMSCs

The femurs and tibiae were removed from immature male Sprague–Dawley rats. The bone marrow plugs were flushed with phosphate-buffered saline (PBS), layered over a Percoll solution (density 1.083), and separated by centrifugation at 2500 RPM for 20 min at room temperature. Mononuclear cells at the interface were recovered and washed twice with PBS. The cells were cultured with complete medium, consisting of DMEM/F12 (1:1) medium supplemented with 10% fetal bovine serum (FBS; Hyclone, Logan, UT), 100 U/mL penicillin, and 100 U/mL streptomycin at 37°C and 5% CO_2_. At confluence, the cells were harvested for passage with 0.25% trypsin containing 0.02% EDTA. All experiments were performed using cells from the third to fifth passage.

BMSC surface expression of CD29, CD34, CD45, and CD90 was analyzed by flow cytometry. Briefly, cells were incubated with the R-phycoerythrin (PE)-labeled antibodies (BD Biosciences, San Jose, California, USA) and their corresponding isotype controls: PE-CD34 (no dilution), PE-CD29 (1:16 dilution), PE-CD45 (1:8 dilution), and PE-CD90 (1:8 dilution). After a 30-min incubation in the dark, the cells were isolated by centrifugation (800 g for 5 min) after which the supernatant was removed. After the cells were washed three times with 2 mL PBS, the cell suspensions were filtered with 200 μm mesh filters to make single cell suspensions. 1× 10^6^Cells were next analyzed for surface marker expression by flow cytometry (Beckman Coulter, Inc., Brea, CA, USA). Samples in which the primary antibody was omitted were used as the gate for the forward scatter (FSC) versus side scatter (SSC).

BMSCs were seeded in 6-well plates at a density of 1 × 10^5^ cells/well. After 24 h, the supernatant in one of the plates was replaced with control DMEM-HG medium supplemented with 10% FBS, 10^-6^ M dexamethasone, 0.5 mM 3-methyl-1-methyl xanthine, 10 μg/mL insulin, and 0.2 mM indomethacin. In the remaining plates, the supernatant was replaced with induction medium comprised of DMEM-HG medium supplemented with 10% FBS, 10^-7^ M dexamethasone, 10 mM beta-glycerophosphate, and 50 mg/mL vitamin C. The medium was replaced every three days. The cells cultured in control medium were stained with Oil red after two weeks; the cells cultured in induction medium were identified by Von Kossa staining after three weeks.

To evaluate the secretion of growth factors, including vascular endothelial growth factor (VEGF), hepatocyte growth factor (HGF), and insulin-like growth factor-1 (IGF-1), by cultured BMSCs, cells were seeded in 6-well plates at a density of 1 × 10^6^ cells/well. After 24 h, the supernatant was replaced with serum-free medium consisting of DMEM/F12 (1:1) medium, 100 U/mL penicillin, and 100 U/mL streptomycin. After three days, the supernatant was collected, and cytokine concentrations were determined using enzyme linked immunosorbent analysis (ELISA) using kits purchased from Uscn (Wu Han, Hubei, China).

### Isolation and identification of GCs

Immature female rats (3–4 weeks) were injected once subcutaneously with 60 IU of pregnant mare serum gonadotropin (Animal Drugs Factory, Huangzhou, Zhejiang, China) to induce the development of multiple antral follicles. After 48 h, the rats were sacrificed, and the ovaries removed. The GCs were isolated by needle puncture under an inverted microscope. After obtaining single cell suspensions, they were washed twice with PBS and cultured with complete medium, including DMEM/F12 (1:1) medium supplemented with 10% FBS, 100 U/mL penicillin, and 100 U/mL streptomycin at 37°C and 5% CO_2_. Cell of the third passage were seeded on microscope slides for immunocytochemistry analysis to confirm their identity. Briefly, single GC suspensions (1 × 10^5^ cells/mL) were fixed with 4% paraformaldehyde and washed three times with PBS (5 min/wash) and then incubated with 0.5% Triton X-100 for 20 min at room temperature. After washing three times with PBS (5 min/wash), cells were incubated with 3% H_2_O_2_ for 5 min at room temperature to eliminate endogenous catalase activity. Nonspecific binding was blocked by incubating the cells will normal goat serum diluted in PBS (1:50) for 20 min at room temperature. Next, samples were incubated with FSHR primary polyclonal antibodies (1:200; Santa Cruz Biotechnology, Santa Cruz, CA) diluted in PBS in a humidified chamber at 4°C overnight. Controls consisted of samples incubated in PBS alone. After washing three times with PBS (5 min/wash), secondary goat anti-rabbit lgG antibodies were added for 60 min at 37°C. After three washes with PBS (5 min/wash), samples were incubated with a horseradish peroxidase-labeled streptomycin solution for 30 min at 37°C prior to color development with the 3,3' Diaminobenzidine (DAB) chromogen. After the reaction was stopped by washing the samples with tap water, the samples were counterstained with hematoxylin for 20 s. Subsequently, the samples were washed, dehydrated, mounted with Permount (Fischer, Fair Lawn, NJ, USA), and analyzed using a Leica TCS-2 microscope (Leica Microsystems Ltd., North Point, Hong Kong, China) equipped with a D1X digital camera (Leica Microsystems Ltd., North Point, Hong Kong, China).

### Effect of BMSCs on GC apoptosis in vitro

Prior to in vitro experiments, 3-(4,5-Dimethylthiazol-2-yl)-2,5-diphenyltetrazolium bromide (MTT) assays (Sigma-Alderich, St Lois, MO, USA) were used to identify the optimal concentrations of cisplatin as well as optimal treatment time without inducing toxicity. GCs (1 × 10^4^ cells/well) in logarithmic growth were seeded onto a 96-well plate and incubated with various concentrations of cisplatin (0, 1.875, 3.25, 7.5, 15, and 30 mg/L). After 24, 48, 72, 96, or 120 h, 20 μL of MTT solution (5 mg/L) was added to each well for 4 h after which 150 μL DMSO was added into each well. The OD value of each well was determined at wavelength 490 nm using a Stat Fax 2100 Microplate Reader (Awareness Technology, INC., Palm City, FL, USA).

Cultured GCs were separated into three groups: an untreated control group (control), a cisplatin (5 mg/L) treatment group (cisplatin), and group co-cultured with BMSCs and treated with 5 mg/L cisplatin (BMSC). GCs were seeded onto 6-well plates at a density of 5 × 10^5^ cells/well. BMSCs were seeded on 6-well Millicell cell culture inserts (Millipore, Billerica, MA, USA) at a density of 2.5 × 10^5^ cells/well. After 24 h, the supernatant was removed. In the control group, the medium was changed to 3 mL serum-free medium (DMEM/F12 medium containing 100 U/mL penicillin and 100 U/mL streptomycin). In the cisplatin group, cells were incubated with 3 mL serum-free medium with cisplatin (5 mg/L). In the BMSC co-culture group, cells were co-cultured with BMSCs and incubated with 3 mL serum-free medium with cisplatin (5 mg/L). After 72 h, the GCs were harvested and analyzed for apoptosis using an annexin V/PI apoptosis detection kit (MultiSciences Biotech, China) according to manufacturer’s protocols and flow cytometry (Beckman Coulter Epics XL Flow Cytometer; GMI, Inc.; Ramsey, Minnesota, USA).

### Real time PCR analysis

GCs from each of the three treatment groups were also harvested to extract RNA. Bcl-2, bax, survivin, c-myc, p21, and GAPDH (housekeeping gene) mRNA expression was examined by real-time PCR using the following primers: and GAPDH mRNA expression was serving as control. The primers sequences are listed as follows: GAPDH (180 bp) sense, 5’-AAGGT CATCCCAGAGCTGAA-3’ and antisense, 5’-CTCAGTGTAGCCCAGGATGC-3’; c-myc (166 bp)sense,5’-TCTCTTCTTCCTCGGACTCG-3’and antisense, 5’-GGTTGCCTCTTTT CCACAGA-3’; p21 (169 bp) sense, 5’-GAGTGCCTTGACGATACAGC-3’ and antisense, 5’-CATGTACTGGTCCCTCATTGC-3’; survivin (180 bp) sense, 5’-GGAGCATAGG AAGCACTCCCCTG-3’ and antisense, 5’-CTCCGGGTCTCCTCGAACTCTT-3’; Bcl-2 (173 bp) sense, 5’-AGTACCTGAACCGGCATCTG-3’ and antisense, 5’-CAGGTATG CACCCAGAGTGA-3’ and Bax (174 bp) sense, 5’-CTGCAGAGGATGATTGCTGA-3’ and antisense, 5’-GATCAGCTCGGGCACTTTAG-3’. PCR amplification conditions consisted of an initial denaturation step of 2 min at 93°C followed by 40 cycles of 95°C for 15 sec and 60°C for 1 min using an ABI 7500 PCR machine (Applied Biosystems; Foster City, CA, USA). The CT values from real time PCR results were analyzed by the 2^-△△CT^ method. The PCR products were validated by running in 120 V × 20 min electrophoresis conditions.

### Effects of BMSCs on GC apoptosis in vivo

Rats reach sexual maturity at 6 to 8 weeks and perimenopause at 10 to 15 months. Female rats (9–10 months) were randomly divided into the following three groups (n = 15 per group): control group, BMSC group, and estrogen group. In addition, 15 adult female rats (3–4 months) were included as the young group. Prior to initiation of the experiment, vaginal smears of the female rats were obtained at 8:30 am every morning to confirm the perimenopausal status of the older rats according to the estrous cycle. Upon confirmation, BMSCs (1–2 × 10^6^ cells in 2 mL) were injected via vena caudalis of rats in the BMSC group; the control and young groups received a 2 mL saline injection. This treatment was repeated after one week. Rats in the estrogen group received 0.158 mg/kg/d nilestriol (Shang Hai New Hua Lian Pharmaceutical CO.LTD, China) for 8 consecutive days by gavage administration. Five rats from each group were randomly sacrificed one and three months after the second transplantation, and their ovaries were removed. The left ovary was fixed in formaldehyde and sectioned for analysis of GC apoptosis using a TUNEL apoptosis assay kit (Roche, Branford, CT). Tissue sections from each animal were examined by microscopy, and at least 100 GCs per field were counted in five randomly selected fields. The GC apoptosis rate was calculated as the number of apoptotic GCs divided by the total number of GCs. Cells were counted independently by two individuals who were blinded to the treatment group. The apoptotic index was defined as the average GC apoptosis rate from counted five randomly selected fields.

### Statistical analysis

Quantitative data are presented as mean ± SD. Differences in gene expression among the three groups (control, cisplatin, and BMSC groups) were detected by one-way analysis of variance (ANOVA) with Tukey’s range test. The GC apoptosis rates were analyzed by two-way ANOVA to evaluate treatment effect (control, BMSC, estrogen) and age as well as time effect (1 month vs. 3 months) along with their interaction. The statistical analyses were performed with SAS software version 9.2 (SAS Institute Inc., Cary, NC). A two-tailed *P*-value < 0.05 indicated statistical significance.

## Competing interests

The authors declare that they have no competing interests.

## Authors’ contributions

JG: guarantor of integrity of the entire study. XG study design. ZL definition of intellectual content. WW: study concepts. LH: literature research. HD: literature research. JC: clinical studies. JL: clinical studies. YF: experimental studies. JW: experimental studies. YM: data acquisition. XC: data acquisition. ZW: data analysis. FH: statistical analysis. SY: manuscript editing. LL: manuscript preparation. FZ: manuscript review. JT: guarantor of integrity of the entire study. All authors read and approved the final manuscript.

## References

[B1] Ross RK Paganini-Hill A Wan PC Pike MC Effect of hormone replacement therapy on breast cancer risk: estrogen versus estrogen plus progestin J Natl Cancer Inst 2000 92 328 332 10.1093/jnci/92.4.328 10675382

[B2] Persson I Weiderpass E Bergkvist L Bergström R Schairer C Risks of breast and endometrial cancer after estrogen and estrogen-progestin replacement Cancer Causes Control 1999 10 253 260 10.1023/A:1008909128110 10482483

[B3] Rossouw JE Anderson GL Prentice RL LaCroix AZ Kooperberg C Stefanick ML Jackson RD Beresford SA Howard BV Johnson KC Kotchen JM Ockene J Writing Group for the Women's Health Initiative Investigators, Risks and benefits of estrogen plus progestin in healthy postmenopausal women: principal results From the Women's Health Initiative randomized controlled trial JAMA 2002 288 321 333 10.1001/jama.288.3.321 12117397

[B4] Palma GA Argañaraz ME Barrera AD Rodler D Mutto AÁ Sinowatz F Biology and biotechnology of follicle development ScientificWorldJournal 2012 2012 938138 22666170 10.1100/2012/938138 PMC3366219

[B5] Orlic D Kajstura J Chimenti S Jakoniuk I Anderson SM Li B Pickel J McKay R Nadal-Ginard B Bodine DM Leri A Anversa PB B**one marrow cells regenerate infarcted myocardium** Nature 2001 410 701 705 10.1038/35070587 11287958

[B6] Fu X Fang L Li X Cheng B Sheng Z Enhanced wound-healing quality with bone marrow mesenchymal stem cells autografting after skin injury Wound Repair Regen 2006 14 325 35 10.1111/j.1743-6109.2006.00128.x 16808812

[B7] Dawn B Bolli R Adult bone marrow-derived cells: regenerative potential, plasticity, and tissue commitment Basic Res Cardiol 2005 100 494 503 10.1007/s00395-005-0552-5 16237509 PMC3685421

[B8] Fu X He Y Xie C Liu W Bone marrow mesenchymal stem cell transplantation improves ovarian function and structure in rats with chemotherapy-induced ovarian damage Cytotherapy 2008 10 353 363 10.1080/14653240802035926 18574768

[B9] Stagg J Immune regulation by mesenchymal stem cells:two sides to the coin Tissue Antigens 2007 69 1 1 9 10.1111/j.1399-0039.2006.00739.x 17212702

[B10] Zhang S Jiang YZ Zhang W Chen L Tong T Liu W Mu Q Liu H Ji J Ouyang HW Zou X Neonatal Desensitization Supports Long Term Survival and Functional Integration of Human Embryonic Stem Cell-derived Mesenchymal Stem Cells in Rat Joint Cartilage without Immunosuppresssion Stem Cells Dev 2012 Epub ahead of print 10.1089/scd.2012.0116 PMC3528094 22788986

[B11] Pittenger MF Mackay AM Beck SC Jaiswal RK Douglas R Mosca JD Moorman MA Simonetti DW Craig S Marshak DR Multilineage potential of adult human mesenchymal stem cells Science 1999 284 143 147 10.1126/science.284.5411.143 10102814

[B12] Chen C Cui J Lu H Wang R Zhang S Shen P Modeling of the role a bax-acctivation switch in the mitochondrial apoptosis decision Biophys Journal 2007 92 4304 4315 10.1529/biophysj.106.099606 PMC1877765 17400705

[B13] Wasiah S Zunino R McBride HM Bax/Bak promote sumoylation of DPP1 and its stable association with mitochondria during apoptotic cell death Cell Biol 2007 177 439 450 10.1083/jcb.200610042 PMC2064824 17470634

[B14] Fu NY Sukumaran SK Yu VC Inhibition of ubiquitin-mediated degradation of MOAP-1 by apoptotic stimuli promotes Bax function in mitochondria Proc Natl Acad Sci USA 2007 104 10051 10056 10.1073/pnas.0700007104 17535899 PMC1877986

[B15] Yi T Baek JH Kim HJ Choi MH Seo SB Ryoo HM Kim GS Woo KM Triehostatin A-mediated upregulation of p21(WAF1) contributes to osteoclast apoptosis Exp Mol Med 2007 39 213 221 10.1038/emm.2007.24 17464183

[B16] Bai J Cederbaum AI Cycloheximide protects HepG2 cels from serum with drawal induced apoptosis by decreasing p53 an d phosphorylated p53 levels J Pharmacol Exp Ther 2006 319 1435 1443 10.1124/jpet.106.110007 16971506

[B17] Zoli W Ulivi P Tesei A Fabbri F Rosetti M Maltoni R Giunchi DC Ricotti L Brigliadori G Vannini I Amadori D Addition of 5-fluomuracil to doxorubicin-paclitaxel sequence increases caspase-dependent apoptosis in breast cancer cell lines Breast Cancer Res 2005 7 R681 R689 10.1186/bcr1274 16168113 PMC1242133

[B18] Zheng QH Ma LW Zhu WG Zhang ZY Tong TJ p21Wafl/Cipl plays a critical role in modulating senescence through changes of DNA methylation J Cell Biochem 2006 98 1230 1248 10.1002/jcb.20838 16514663

[B19] Cao X Bennett RL May WS C-myc and caspase-2 are involved in activating bax during cytotoxic drug-induced apoptosis Biol Chem Biol 2008 283 14490 14496 10.1074/jbc.M801107200 PMC2386933 18375382

[B20] Takehara Y Yabuuchi A Ezoe K Kuroda T Yamadera R Sano C Murata N Aida T Nakama K Aono F Aoyama N Kato K Kato O The restorative effects of adipose-derived mesenchymal stem cells on damaged ovarian function Lab Invest 2012 [Epub ahead of print] 10.1038/labinvest.2012.167 PMC3561594 23212100

[B21] Zhang J Li Y Zheng X Gao Q Liu Z Qu R Borneman J Elias SB Chopp M Bone marrow stromal cells protect oligodendrocytes from oxygen-glucose deprivation injury J Neurosci Res 2008 86 1501 1510 10.1002/jnr.21617 18214988 PMC2593416

[B22] Mahmood A Lu D Chopp M Intravenous administration of marrow stromal cells (MSCs) increases the expression of growth factors in rat brain after traumatic brain injury J Neurotrauma 2004 21 33 39 10.1089/089771504772695922 14987463

[B23] Wang L Zhang Z Wang Y Zhang R Chopp M Treatment of stroke with erythropoietin enhances neurogenesis and angiogenesis and improves neurological function in rats Stroke 2004 35 1732 1717 10.1161/01.STR.0000132196.49028.a4 15178821

[B24] Xu M Uemura R Dai Y Wang Y Pasha Z Ashraf M In vitro and in vivo effects of bone marrow stem cells on cardiac structure and function J Mol Cell Cardiol 2007 42 441 448 10.1016/j.yjmcc.2006.10.009 17187821 PMC1899533

[B25] Neulen J Wenzel D Hornig C Wünsch E Weissenborn U Grunwald K Büttner R Weich H Poor responder-high responder:the importance of soluble vascular endothelial growth factor receptor 1 in ovarian stimulation protocols Hum Reprod 2001 16 621 626 10.1093/humrep/16.4.621 11278207

[B26] Ginther OJ Bergfelt DR Beg MA Meira C Kot K In vivo effects of an intrafolicular injection of insulin-like growth factor 1 on the mechanism of follicle deviation in heifers and mares Biol Reprod 2004 70 99 12954722 10.1095/biolreprod.103.021949

[B27] Depalo R Nappi L Loverro G Bettocchi S Caruso ML Valentini AM Selvaggi L Evidence of apoptosis in human primordial and primary follicles Hum Reprod 2003 18 2678 2682 10.1093/humrep/deg507 14645191

[B28] Ito M Harada T Tanikawa M Fujii A Shiota G Terakawa N Hepatocyte growth factor and stem cell factor involvement in paracrine interplays of theca and granulosa cells in the human ovary Fertil Steril 2001 75 973 979 10.1016/S0015-0282(01)01747-2 11334911

[B29] Uzumcu M Pan Z Chu Y Kuhn PE Zachow R Immunolocalization of the hepatocyte growth factor (HGF) system in the rat ovary and the anti-apoptotic effect of HGF in rat ovarian granulosa cells in vitro Reproduction 2006 132 291 299 10.1530/rep.1.00989 16885537

